# Seroprevalence and risk factors associated with toxoplasmosis and hydatidosis among the butchers of Tabriz city, the northwest of Iran: a case control study

**DOI:** 10.1186/s12995-024-00427-4

**Published:** 2024-07-22

**Authors:** Zahra Amiri, Shahram Khademvatan, Tohid Kazemi, Elham Yousefi

**Affiliations:** 1 Cellular and Molecular Research Center, Cellular and Molecular Medicine Institute, Department of Medical Parasitology and Mycology, Urmia University of Medical Sciences, Urmia, Iran; 2 Department of Immunology, School of Medicine Immunology Research Center, Stem Cells and Regenerative Medicine Institute, Tabriz University of Medical Sciences, East Azerbaijan, Iran

**Keywords:** Butchers, Frequency, Hydatidosis, Iran, Risk factors, Toxoplasmosis

## Abstract

**Introduction:**

Occupation plays an important role in the spread of infectious diseases in humans. Toxoplasmosis and hydatidosis are world-wide diseases with different routes of transmission. This study aimed to investigate the prevalence of toxoplasmosis and hydatidosis and risk factors associated with these diseases among the butchers of Tabriz City, the northwest of Iran.

**Methods:**

In this case-control study conducted in Tabriz city in 2023, 250 serum samples were collected from butchers (*n* = 125) and outpatients referred to Imam Reza Hospital (*n* = 125) and. The ELISA test was used to identify IgG and IgM antibodies against toxoplasmosis and IgG antibodies against hydatidosis. The results were analyzed by statistics tests using SPSS v. 16 software. Risk factors’ association was tested using Chi square or logistic regression analysis.

**Results:**

The results indicated that 66/125 (52.8%) cases and 40/125 controls (32%) were positive for toxoplasmosis IgG antibody. Also, 5/125 (4%) and 1/125 (0.8%) were positive for toxoplasmosis IgM antibody in the case and control groups, respectively. In addition, 10/125 people (8%) were positive for anti-hydatidosis IgG antibody in the case group, while no positive cases were found in the control group. The main risk factors for toxoplasmosis were age (OR: 1.014), education level (OR: 0.638), and work experience(OR: 1.695), these factors for hydatidosis included age and education level (OR: 1.765 and 0.271) respectivily.

**Conclusions:**

Our results suggest the high prevalence of toxoplasmosis and in butchers of Tabriz, which required special attention and basic measures. Moreover, the prevalence of hydatidosis IgG antibodies also requires more attention to be focused on breaking the transmission and reducing the infection.

## Background

Today, occupational infections remain a major health concern all over the world [1]. Harmful factors in the work environment can result in various diseases, including infectious diseases, which are mostly chronic [2]. Knowing the cause of a disease is the first and most essential step in the prevention of work-related diseases. Examination of clinical symptoms and evaluation of any changes in people’s health, together with conducting periodic examinations and epidemiological studies, could be efficient strategies for the diagnosis of these diseases [1, 2].

One of the most significant risk factors of parasitic diseases is the risky nature of different jobs, which contribute to the transmission of disease through the direct contact with the infectious sources that are an integral part of some jobs [3]. Toxoplasmosis and hydatidosis, known parasitic diseases, can cause serious complications in the host, if ignored. The main risk factors for toxoplasmosis and hydatidosis in butchers include slaughtering practices and contact with raw meat as a potential source of infection. Butchers have a close contact with livestock, their visceral, and raw or infected meat; therefore, the probability of encountering them with the parasitic diseases, including toxoplasmosis and hydatidosis, increases [4, 5]. Toxoplasmosis is a prevalent disease between animals and humans with a global distribution and is caused by the parasite *Toxoplasma gondii*. In a general population, toxoplasmosis can remain asymptomatic and give rise to lymphadenopathy and flu-like symptoms, but it can be lethal in immunosuppressed patients [6, 7]. The prevalence rate of infection with this parasite in different countries and even different regions of a country varies based on factors such as age, sex, occupation, and nutrition type [8, 9]. The seroprevalence of toxoplasmosis in France, America, Nigeria, and South Korea has been reported to be 47%, 6%, 23.9%, and 6.7%, respectively [10]. In Iran, the overall prevalence of toxoplasmosis is high, and various risk factors are involved in the high prevalence of this disease in the country [11]. Humans can be infected with toxoplasmosis in multiple ways, including consuming raw or undercooked meat, unwashed fruits and vegetables, direct contact with oocysts in cat feces, blood transfusions, and transplants [12–14]. Butchers are constantly in close contact with animals and their products after slaughter and among the first people who encounter *Toxoplasma* tissue cysts in meat; therefore, they are considered a high-risk group for this disease [5].

Hydatidosis is another parasitic disease regarded as an occupational health problem for dog owners, sheep farmers, and butchers in endemic areas [4]. It is an important zoonotic disease caused by the tapeworm *Echinococcus granulosus* [15]. The life cycle of this parasite comprises two hosts, a definitive carnivore host and an intermediate host, in which the adult form of the parasite and the larval stage develop, respectively. Exposure to parasite eggs that are excreted from definitive host feces acts a prominent role in disease transmission [4, 15]. Hydatidosis occurs globally; however, the main endemic areas for this disease are India, Middle East, China, Australia, South America, France, and North Africa. The reported annual incidence of hydatidosis in humans is around 1 million with expenses amounting to $ 3 billion every year [16]. The total estimated prevalence of human hydatid cyst infection in Iranian individuals was 2.4%, which is chiefly localized in the southern parts of the country [17]. Age, occupation, place of residence, the presence of dogs in a residential place, unhygienic slaughtering practices, consumption of unwashed vegetables are the most important risk factors of hydatidosis [18]. Among these factors, occupation is an important determinant in the epidemiology of hydatidosis. Butchers are certainly at potential risk of being infected with this infection [16]. Slaughterhouse workers are at risk of acquiring hydatidosis through direct contact with infected animals, as well as through exposure to contaminated water or soil in the workplace. Inadequate hygiene practices and lack of proper protective equipment can also increase the risk of transmission.

Occupation and continuous contact with the source of contamination have a crucial role in the spread of infectious diseases in humans. Considering the harmful impact of toxoplasmosis and hydatidosis on human health and their prevalence in Iran and given the lack of sufficient information on the risk factors of these two parasitic infections, including the risk of occupational exposure to raw meat, this study was undertaken to determine the prevalence and risk factors associated with toxoplasmosis and hydatidosis among the butchers of Tabriz City, the northwest of Iran.

## Methods

### Study population and sampling

The present study included 125 butchers from all areas of Tabriz City (case group) and 125 outpatients referred to Imam Reza Hospital in the same city (control group).The samples collected between March and December of 2023. The sample size was calculated using StatCalc in Epi Info version 7.2.1.0. Using a prevalence, from a similar study Daniela Almeida), (P1 = 0.728, P2 = 0.601, α = 0.05, β = 0.8) [19].

total sample size of tow group 250 was obtained [19]. The city divided into 5 central, northern, southern, eastern, and western regions, sampling an equal number was done from among these neighborhoods. After written informed consents were obtained from all the participants, 5 ml of the blood was collected. Then each butcher filled a checklist containing demographic information and education level, as well as a history of hand injury and using personal protective equipment. Serum samples were kept at -20 °C until experimental assay.

### Detection of Anti-*Toxoplasma Gondii* and hydatid cystic antibodies

ELISA technique was use to measure antibodies against toxoplasmosis and hydatidosis using anti-*toxoplasma* IgG, IgM, and anti-*Echinococcus* IgG kits from Pishtaz Teb (Cat no. LOT98003, LOT01003, LOT97001, respectively; Tehran, Iran). Both kits applied indirect ELISA assay, which was performed based on manufacturers’ instructions. In brief, serum samples were first added to an antigen-coated plate, and then after a definite incubation time, HRP (Horse Radish Peroxidase) conjugation was performed. In each step, the plate was washed to remove excess unbound material. Following the addition of the chromogenic substrate to develop color change, OD (Optical Density) values were read at 450 and 630 nm in a microplate spectrophotometer (Lovibond, Germany). Negative and positive controls were provided by the above-mentioned kits. Cut-off and index values to determine negative and positive results were calculated based on OD values according to the kit protocol. The sensitivity and specificity of the aforesaid kits were reported as 91% and 96% for echinococcus and ~ 100% for toxoplasma according to the kit manufacturer.

### Data analysis

Statistical analysis was performed using IBM SPSS version 16. The chi-square with Yate’s correction test for homogeneity of proportions was used to calculate significant differences in anti-*T. gondii* IgG and IgM and anti-echinococcus IgG seroprevalence between the two study groups. Binary and multinomial logistic regression analyses were carried out to determine which of the variables (region, gender, age, and years of work) were significantly associated with the detection of anti-*T. gondii* IgG and IgM, as well as anti-echinococcus IgG among the butchers. A p value less than 0.05 was considered statistically significant.

## Results

Of 125 butchers in this study, 36 (28.6%) were in the age range of 30–40 years, 57 (45.6%) had a diploma degree, and 84 (67.2%) had been working as a butcher for more than 10 years (Table 1). The butchers had a mean age of 46.2 ± 12.29 years. From 125 butchers’ sera samples tested, 66 (52.8%) were positive for the existence of anti-*T. gondii* IgG antibodies and 5 (4%) were seropositive for anti-*T. gondii* IgM antibodies, suggesting a chronic and acute toxoplasmosis, respectively. In addition, of 125 control samples, 40 (32%) were positive for the existence of anti-*T. gondii* IgG antibodies, and only 1 (0.8%) was positive for the existence of *T. gondii* IgM antibody. Chi-square test showed a significant difference in anti-*T. gondii* IgG seroprevalence between the case and control groups (*p* = 0.001), but this trend for *T. gondii* IgM antibody between the two study groups was reverse (*p* = 0.098). Comparing the frequency of hydatidosis in the case group with that of the control group showed that 10 butchers (8%) were positive for hydatidosis IgG antibody, but no positive cases were observed in the control group. The chi-square test results indicated that the difference in hydatidosis IgG seroprevalence between the study groups was statistically significant (*p* = 0.002) (Table 2).

**Table 1:** Demographic information of butchers in this

Demographic variables	Frequency (%)
**Age (y)**	
< 20	5 (4)
20–30	18 (14.4)
30–40	36 (28.6)
40–50	32 (25.6)
50–60	28 (22.4)
> 60	6 (4.8)
**Education**	
Below high school	11 (8.8)
High school	43 (34.4)
Diploma	57 (45.6)
Bachelor’s degree	11 (8.8)
Post graduated	3 (4.2)
**Work experience (y)**	
< 1	6 (4.8)
1–5	17 (13.6)
5–10	18 (14.4)
< 10	84 (67.5)

**Table 2:** Distribution of positive and negative anti *T. Gondii* IgG and IgM and anti-hydatidosis IgG antibodies from serum samples of study groups

Groups	Toxoplasmosis				Hyadatid Cyst		Total
	IgG		IgM				
	Positive no. (%)	Negative no. (%)	Positive no. (%)	Negative no. (%)	Positive no. (%)	Negative no. (%)	
Case	66 (52.8)	59 (47.2)	5 (4)	120 (96)	10 (8)	115 (92)	125
Control	40 (32)	85 (68)	1 (0.8)	124 (92)	0 (0)	125 (100)	125
Total	106 (42.4)	144 (57.6)	6 (2.4)	244 (94)	10 (4)	240 (96)	250

The results of this study exhibited that 3 (4.4%) butchers were infected with both toxoplasmosis and hydatidosis simultaneously, though there was no significant association between toxoplasmosis and hydatidosis positivity. To examine the risk factors of toxoplasmosis among butchers, we assessed the total positive antibodies (IgG and IgM). The association between the detection of anti-*T. gondii* antibodies in butchers and variables was evaluated by chi-square test and binomial logistic regression (univariate and multivariate). The results of chi-square test showed four statistically significant risk factors, including soil contact (*p* < 0.001; OR = 6.5), cat contact (*p* = 0.033; OR = 2.22), consuming raw or undercooked meat (*p* = 0.031; OR = 5.98), and consuming unboiled milk (*p* = 0.007; OR = 4.03), for toxoplasmosis infection in butchers (Table 3).

**Table 3:** Seroprevalence of toxoplasmosis based on chi-square analysis of risk factors

Variables	*N* (%)	Toxoplasmosis		*p* value	OR
		Positive no. (%)	Negative no. (%)		
**Soil contact**					
Yes	33 (25.1)	27 (81.82)	6 (18.18)	< 0.001	5.6
No	92 (74.9)	41 (44.57)	51 (55.43)		
**Cat contact**					
Yes	50 (39.15)	33 (66)	17 (34)	0.033	2.22
No	75 (60.85)	35 (46.67)	40 (53.33)		
**Using gloves during work**					
Yes	27 (21.95)	12 (44.45)	15 (55.55)	0.241	0.6
No	98 (78.05)	56 (57.14)	42 (42.86)		
**Consuming raw or undercooked meat**					
Yes	6 (4.4)	6 (100)	0 (0)	0.031	5.98
No	119 (95.6)	62 (52.1)	57 (47.9)		
**Injuring hands during work**					
Yes	113 (90.65)	59 (52.21)	54 (47.79)	0.132	0.36
No	12 (8.35)	9 (75)	3 (25)		
**Washing hands before eating**					
Yes	115 (91.9)	63 (54.78)	52 (45.22)	0.771	1.21
No	10 (8.1)	5 (50)	5 (50)		
**Exposing meat and blood to face during work**					
Yes	93 (73.8)	55 (59.14)	38 (40.86)	0.07	2.12
No	32 (26.2)	13 (40.62)	19 (59.38)		
**Eating and drinking during work**					
Yes	111 (88.85)	60 (54.05)	51 (45.95)	0.827	0.88
No	14 (11.15)	8 (57.14)	6 (42.86)		
**Consuming unboiled milk**					
Yes	24 (18.35)	19 (79.17)	5 (2.83)	0.007	4.03
No	101 (81.65)	49 (48.51)	52 (51.49)		
**Consuming unwashed vegetable**					
Yes	11 (8.8)	6 (54.55)	5 (45.45)	0.992	1.01
No	114 (91.2)	62 (54.39)	52 (45.61)		

The results of the same test in Table 4 demonstrated a significant correlation between the positive serology of anti-hydatidosis antibody and soil contact (*p* = 0.012; OR = 4.89), dog contact (*p* ≤ 0.001; OR = 15.43), and consuming unwashed vegetables (*p* < 0.001; OR = 17.18). Based on the binomial logistic regression (univariate analysis), there was an association between work experience (*p* = 00.11; OR = 763.1) and anti *T. gondii* antibodies seropositivity (IgG and IgM). Multivariate analysis also showed that education level (*p* = 0.094; OR = 0.63), age (*p* = 0.947; OR = 1.014), and work experience (*p* = 0.011; OR = 1.69) were the risk factors involved in toxoplasmosis infection (Table 5). However, education level (*p* = 0.005; OR = 0.187) and age (*p* = 0.024; OR = 2.471) were found to be related to anti-hydatidosis IgG seropositivity, as represented by the binomial univariate logistic regression. Based on the multivariate analysis, the only risk factor for anti-hydatidosis IgG seropositivity was level of education, with a p value of 0.043 and an OR of 0.271 (Table 5).

**Table 4:** Seroprevalence of hydatidosis based on chi-square analysis of risk factors

Variables	*N* (%)	Hydatidosis seroprevalence		*p* value	OR
		Positive no (%)	Negative no (%)		
**Soil Contact**					
Yes	33 (41.75)	6 (18.18)	27 (81.82)	0.012	4.89
No	92 (58.25)	4 (4.35)	88 (95.65)		
**Dog Contact**					
Yes	12 (28.05)	5 (41.67)	7 (58.33)	< 0.001	15.43
No	113 (71.95)	5 (4.42)	108 (95.58)		
**Washing hands before eat and drink**					
Yes	115 (95.65)	10 (8.7)	105 (91.3)	0.42	1.05
No	10 (4.35)	0 (0)	10 (100)		
**Eating and drinking during work**					
Yes	111 (93.9)	10 (9.01)	101 (90.99)	0.601	1.5
No	14 (6.1)	0 (0)	14 (100)		
**Consuming unwashed vegetables**					
Yes	11 (27.6)	5 (45.45)	6 (54.55)	< 0.001	18.17
No	114 (72.4)	5 (95.61)	109 (95.61)		

**Table 5:** Univariate and multivariate analysis of risk factors for toxoplasmosis and hydatidosis

Variables	Toxoplasmosis				Hydatidosis			
	Multivariate		Univariate		Multivariate		Univariate	
	*p* value	OR	*p* value	OR	*p* value	OR	*p* value	OR
Work experience	0.049	1.695	0.011	1.763	0.907	0.92	0.342	0.615
Education level								
Under Diploma	0.094	0.638	0.041	0.607	0.043	0.271	0.005	0.187
Diploma and upper	-	-	-	-	-	-	-	-
Age	0.947	1.014	0.037	1.4	0.352	1.765	0.024	2.471

## Discussion

A disease or disorder that occurs due to an exposure to risk factors arising from work activities is considered an occupational disease [1]. If ignored, these diseases can cause irreparable damage to humans [2]. Butchers who are in close contact with livestock and raw meat are at particular risk of zoonotic parasitic infections such as toxoplasmosis and hydatidosis [4, 5].

This study observed a significant higher anti-*T. gondii* IgG seroprevalence in the case (52.8%) than control (32%) group (*p* = 0.001), but seropositivity for anti-*T. gondii* IgM in the case group was insignificant, as compared to the control group (*p* = 0.098). A possible reason for the high prevalence of anti-*T. gondii* IgG could be the butchers’ lack of knowledge about the nature of parasitic infections and occupational diseases and the route of their transmission.

In the present study, two of several important risk factors for toxoplasmosis in butchers were age and work experience. Owing to the longer period of exposure to risk factor, raise in the probability of toxoplasmosis among butchers was not predictable with increasing age and work experience. In our study, butchers had a constant and easy access to meat and their preference was to eat uncooked meat; therefore, these two factors became significant risk factors in the occurrence of toxoplasmosis in butchers. Other seroepidemiological studies have suggested similar findings in people occupationally exposed to livestock. A study from Portugal has reported an anti-*T. gondii* IgG seroprevalence of 75.8% for butchers and slaughterhouse workers compared to 60.1% in the control group. Similar to our study, researchers have identified that the aging of butchers is a reason for this high prevalence of toxoplasmosis [19]. This observation has also been found in the Central India, with an anti-*T. gondii* IgG seroprevalence of 48.4% in slaughterhouse workers and butchers vs. 6.6% of the general population. Some major reasons for the high prevalence of toxoplasmosis among butchers aged > 30 years are cat contact, wearing gloves, and contact with soil [20]. In another similar research, Alvarado-Esquivel and colleagues introduced the years spent as a butcher as a potential risk factor for toxoplasmosis [21]. In additional similar study conducted among the butchers of Sanandaj City, the west of Iran, eating while working and wearing gloves were the most significant risk factors for toxoplasmosis in butchers [5]. A study in the Northeast of Iran in Mashhad City among slaughterhouse workers demonstrated increasing age of workers and work duration as the potential risk factors for toxoplasmosis [4]. In a comparable study conducted in Khuzestan, the southwest of Iran, on butchers, Mardani et al. presented 48.8% anti-*T. gondii* IgG in butchers vs. 28.8% in the control group, and attributed this high prevalence to the work experience of the butchers, which was in line with our results [22]. The common point of our study and all the mentioned studies is the high prevalence of anti-*T. gondii* IgG antibodies among butchers compared to controls. Although our results are in conformity with various studies, some researchers have shown different conclusions. In a study conducted by Thiong’o et al. in Kenya, no significant differences were found in the seropositivity of *T. gondii* IgG antibody among slaughterhouse workers and butchers compared to the general population [23], likely due to applying methods with variable sensitivities. Despite all the aforesaid interpretations, the relative importance of the risk factors varies between cities and countries due to differences in cultural patterns and environmental factors. In general, the prevalence of the infection varies depending on the epidemic area, socio-cultural behaviors, and geographical and climatic factors.

To our knowledge, this is the first case-control study reporting the prevalence of anti-hydatidosis IgG in butcher in the northwest of Iran. In this study, we indicated significantly higher anti-hydatidosis IgG antibody seroprevalence in butchers (10/8%) of Tabriz City compared to the controls (0/0%). Among risk factors investigated herein, education level, age, contact with dogs, contact with soil, and consuming unwashed vegetables were statistically significant factors for hydatidosis seroprevalence in butchers of the stated city. This outcome was predictable given that with increasing the age of butchers, the risk of hydatidosis raises because of the slow development of hydatid cyst in intermediate hosts and humans. In addition, the lower level of education in butchers, the lower awareness of contacting parasitic and infectious diseases and the higher exposure to the infections. A number of studies have confirmed our results. In a study conducted in Pakistan, Alvi et al. found that 9.61% of the samples were seropositive for anti-IgG hydatidosis in butchers. They have also observed that contact with dog, age > 30 years, lower education levels, and work experience for > 10 are the main reasons for the high prevalence of hydatidosis in butchers [16] A similar investigation among slaughterhouse workers revealed a 5.5% anti-IgG hydatidosis seroprevalence in Mashhad [4]. In another study in India, the prevalence of hydatidosis among butchers who were arguably at the potential risk of being infected with the disease was reported as 15.4% [24]. Asadi’s investigation in Urmia, the northwest of Iran, the same as our study, showed that the consumption of unwashed vegetables is the main risk factor for hydatidosis [18]. The study by Sakhaei et al. in Jolfa County, the Northwestern Iran, displayed that consumption of unwashed vegetables and contact with dogs were more seropositive for hydatidosis [15]. Results of our study displayed that occupation remains a significant factor in the epidemiology of hydatidosis and suggested butchers as one of the main occupational risk groups for hydatidosis. Of note, anti-hydatidosis IgG seroprevalence in the control group of this study (0/0%) was lower than that of some other studies [15, 25], mainly due to enhancing knowledge of people about zoonotic diseases and prevention measures, or it is likely related to the large number of their samples.

Butchers were positive and negative for both parasitic infections, and no significant relationship was observed between toxoplasmosis and hydatidosis positivity. This result of our study is consistent with those of Yousefi et al.’s investigation in Mashhad that reported 3 (3.3%) and 36 (39.6%) slaughterhouse workers were respectively positive and negative for both infections, and there was no significant relation between toxoplasmosis and cystic echinococcosis positivity [4]. Butchers are among at-risk occupations regarding parasitic infections, including toxoplasmosis and hydatidosis. This group is in close contact with meat and livestock as a source of contamination. Therefore, butchers should be given the necessary equipment and training to change the process of preparing meat for sale from manual and traditional to industrial and automatic methods, which will reduce the exposure to raw meat. Owing to financial and time limitations, we performed limited sensitivity tests to confirm positive cases. Additional studies with more sensitivity tests could manage this concern.

## Conclusions

The findings of this study reveal a high prevalence of toxoplasmosis and hydatidosis anti-IgG antibodies in butchers of Tabriz City. Considering the high incidence of these two important parasitic infections in butchers, it is recommended to inform them about the risk of parasitic and occupational infections. Moreover, more control and supervision are needed to be performed on the preparation and distribution of raw meat.

## Data Availability

No datasets were generated or analysed during the current study.
